# Supplementary material for: Integrative analysis of genomic and epigenomic regulation reveals miRNA mediated tumor heterogeneity and immune evasion in lower grade glioma
Source: Commun Biol. 2024 Jul 6;7:824. doi: 10.1038/s42003-024-06488-9 (PMC11227553; doi:10.1038/s42003-024-06488-9)
Supplement: Supplementary file 2 — Supplemental information [file 42003_2024_6488_MOESM2_ESM.pdf]

Supplemental information

Integrative analysis of genomic and epigenomic regulation reveals microRNA mediated tumor heterogeneity and immune evasion in lower grade glioma

Supplementary Figures

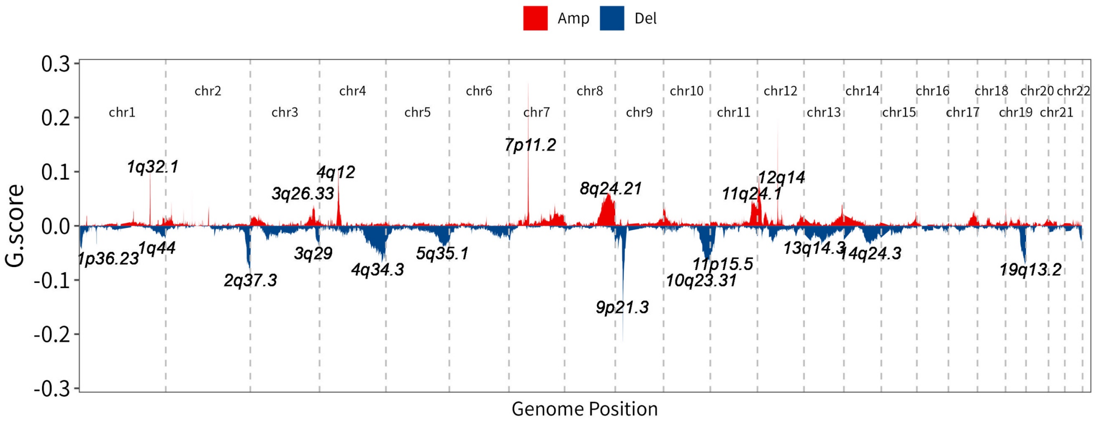

Supplementary Figure 1: Global view of copy number variation for LGG. The copy number amplitude was indicated by the G-scores that calculated by GISTIC2, amplifications are indicated as red and deletions are indicated as blue.

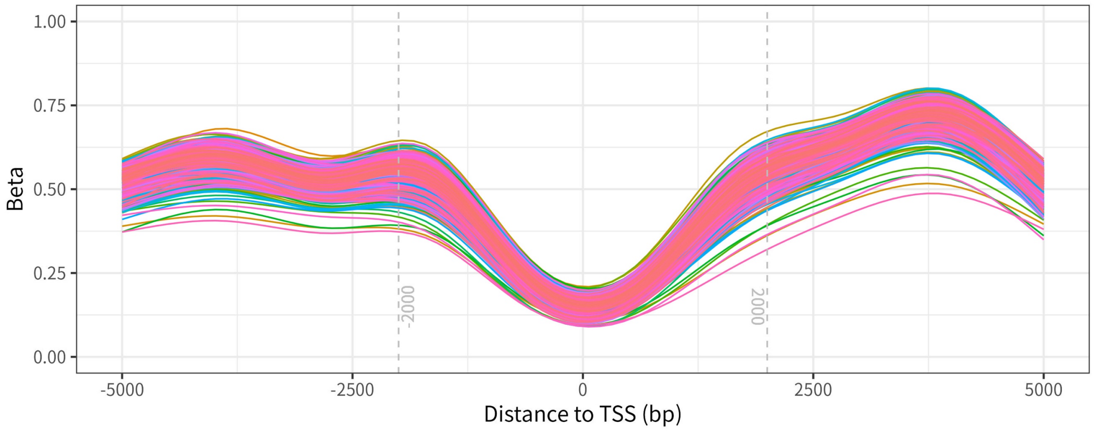

Supplementary Figure 2: Metaplot of CpG methylation levels around miRNA transcription start site for all LGG samples. The genomic region between [-2000, 2000] is indicated by the vertical dotted lines. The curves with different color was used to indicate different samples.

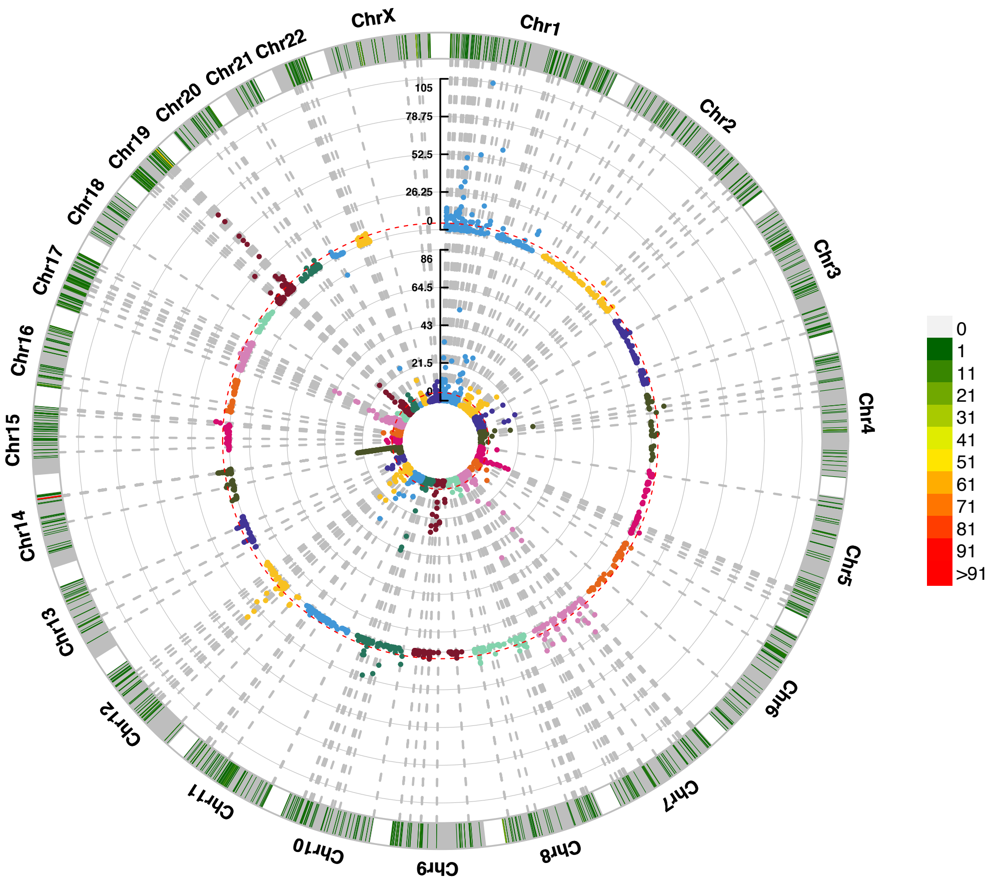

Supplementary Figure 3: Genome-wide landscape of miRNA loci with associations between expression and CNV/DNAm. Each dot represents a miRNA loci, chromosomes were shown in different colors. Y-axis within each circle showed the P-value in corresponding association analysis. Association analysis results for DNAm and CNV were plotted from inside to outside.

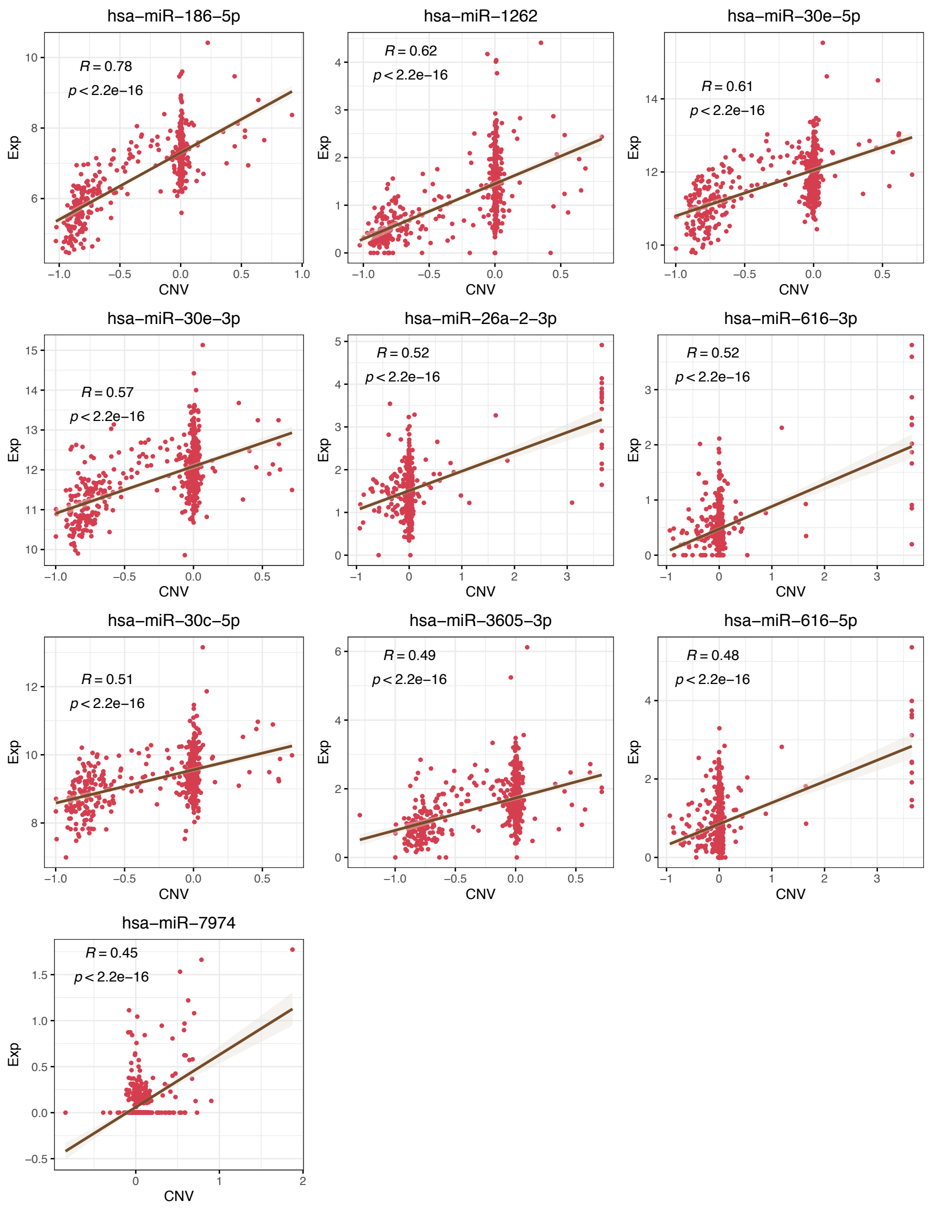

Supplementary Figure 4: Scatter plot of top rank miRNAs with significant correlation between expression and copy number variation. P-values are generated from Pearson correlation analysis.

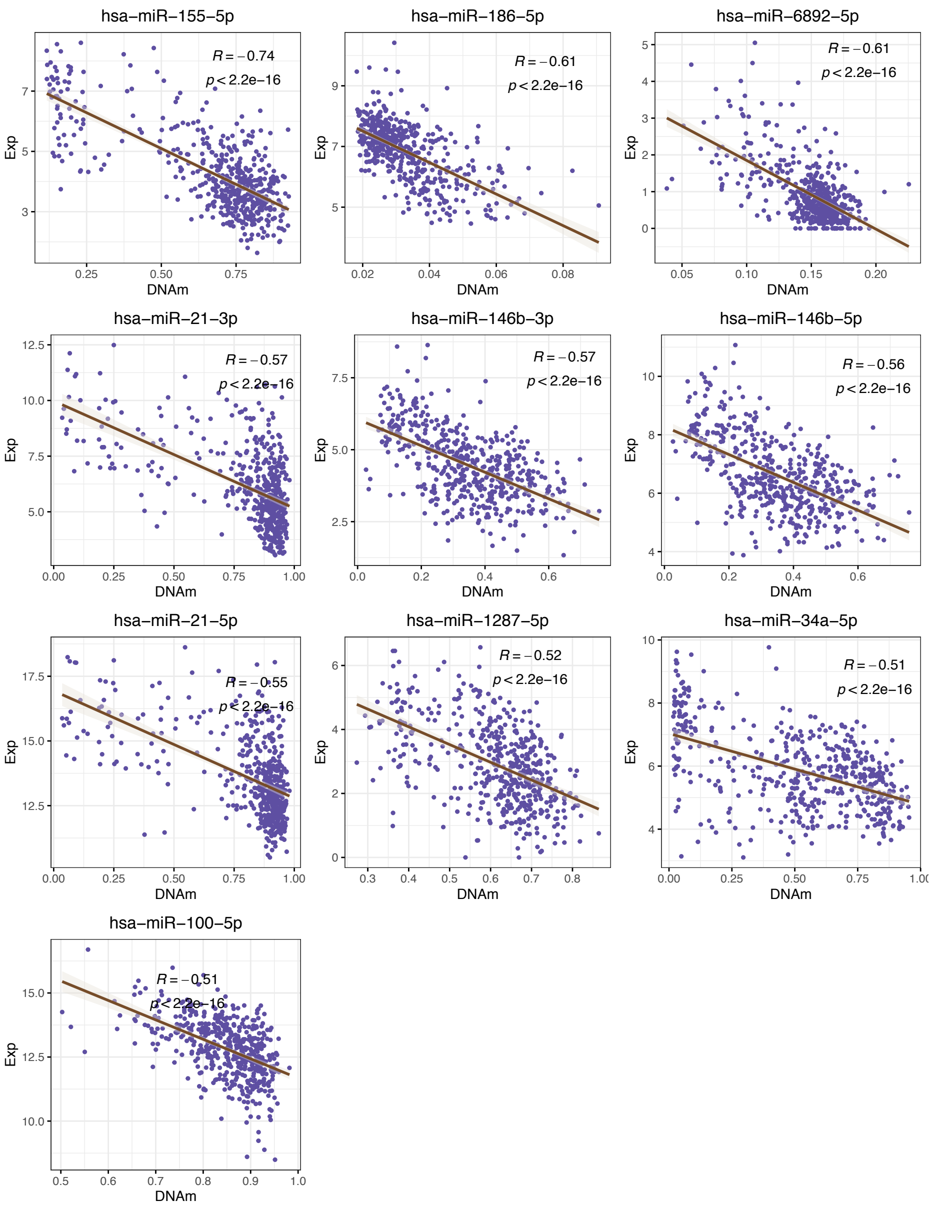

Supplementary Figure 5: Scatter plot of top rank miRNAs with significant correlation between expression and DNA methylation. P-values are generated from Pearson correlation analysis.

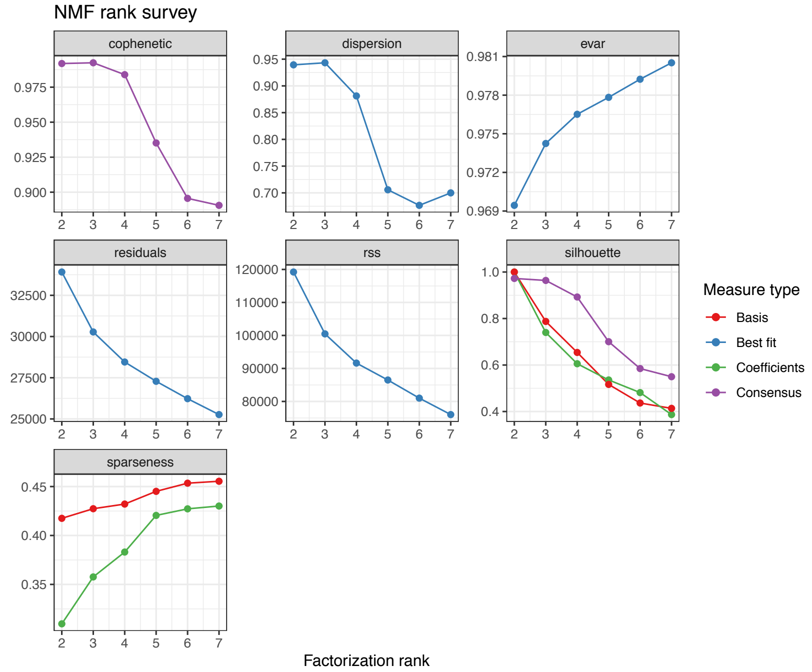

Supplementary Figure 6: NMF clustering analysis for DNAm-miRs. The NMF clustering method was used to evaluate the clustering number k as 2-7. Cophenetic, dispersion, evar, residuals, rss, silhouette, and sparseness values were evaluated to find the optimal clustering number by combining these values in a consensus matrix.

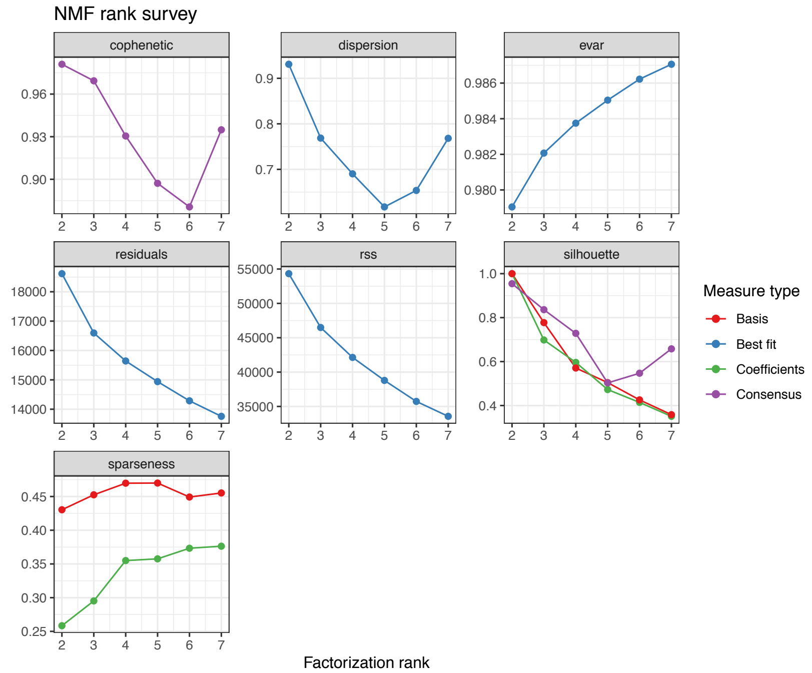

Supplementary Figure 7: NMF clustering analysis for CNV-miRs. The NMF clustering method was used to evaluate the clustering number k as 2-7. the optimal clustering number was selected as before.

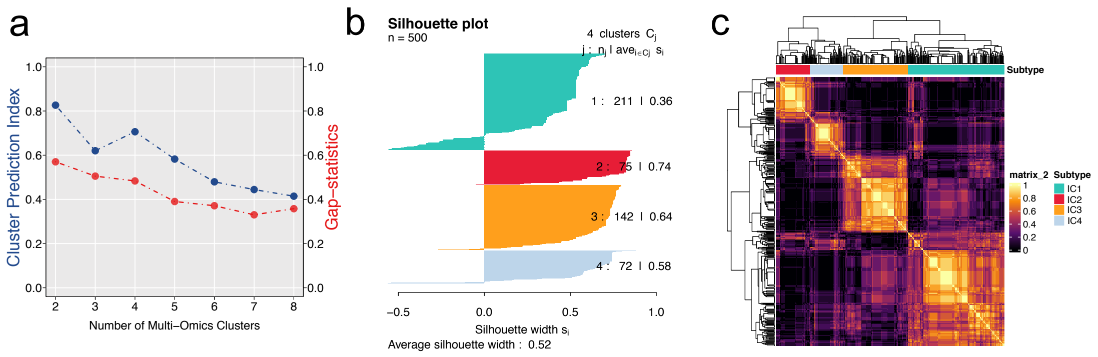

Supplementary Figure 8: Consensus ensemble clustering based on multi-omics data. a). Determination of optimal clustering number based calculating Cluster Prediction Index (blue dots) and Gaps-statistics (red dots); b). Silhouette plot shows the consistency for the predictions among different clustering methods; c). Consensus heatmap based on prediction results from 10 integrative clustering methods.

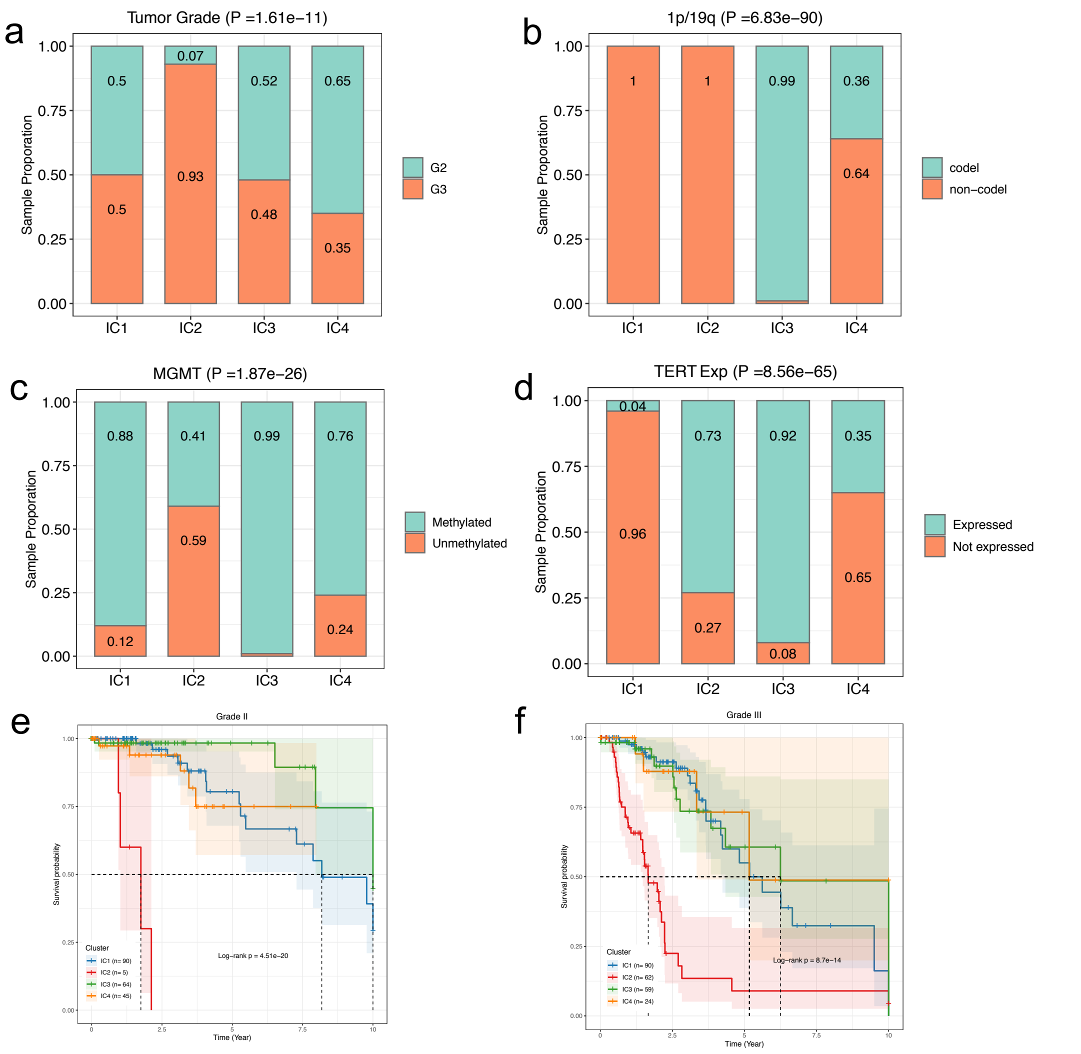

Supplementary Figure 9: Comparison of the clinical characteristics among clusters of LGG patients. a). Barchart showing that the IC2 cluster has significantly more patients with grade 3 than that of others; b). IC1 and IC2 clusters have significantly more patients with 1p/19q codeletion; c). IC2 cluster has significantly more patients with MGMT promoter methylation; d). IC and IC4 clusters have significantly more patients with TERT expression; e). Kaplan-Meier survival curve of different clusters in grades II; f). Kaplan-Meier survival curve of different clusters in grades III.

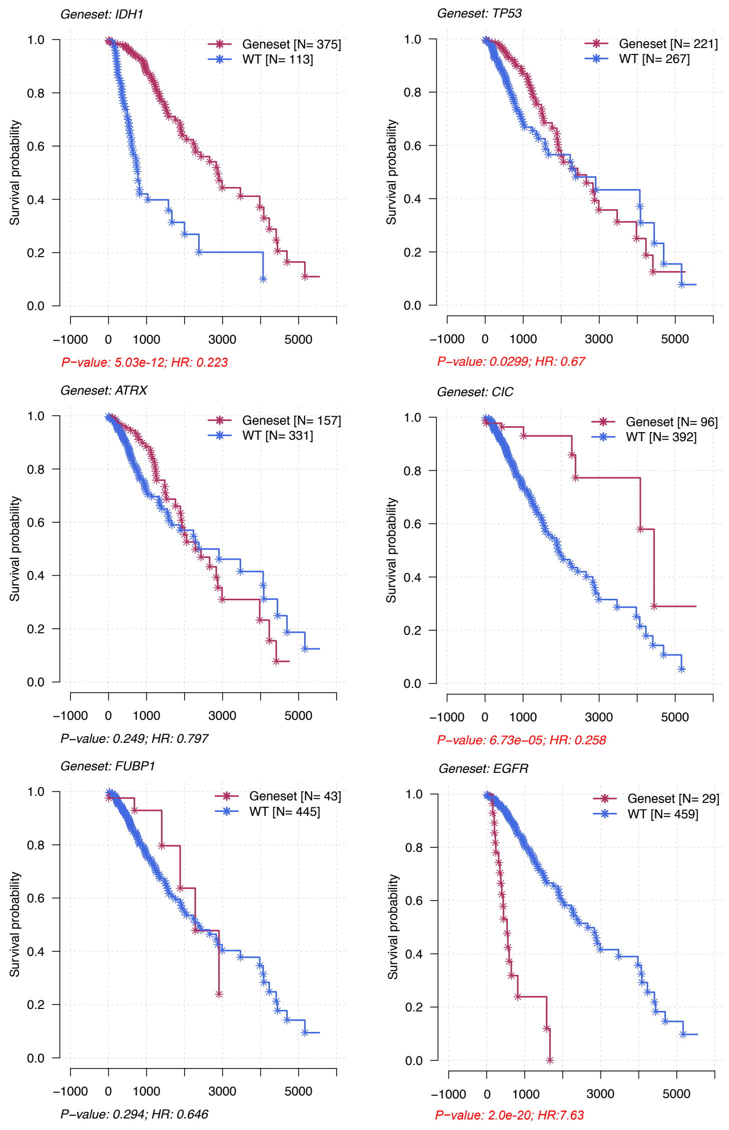

Supplementary Figure 10: Kaplan–Meier plot of survival for patients with different mutational status for six genes of *IDH1*, *TP53*, *ATRX*, *CIC*, *FUBP1* and *EGFR*, respectively. The log-rank test p-values and Hazard Ratio (HR) were indicated.

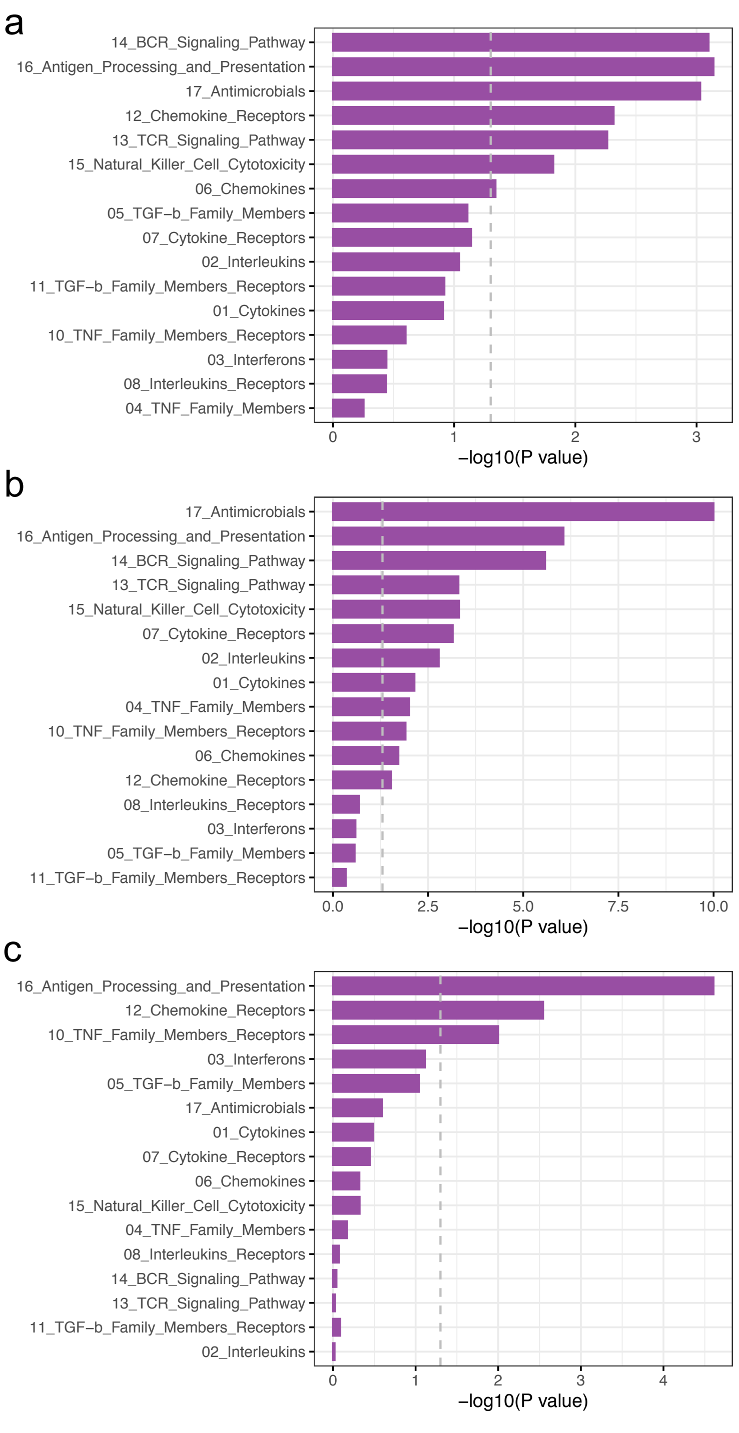

Supplementary Figure 11: Bar plot showing immune related pathways enriched by genes differentially expressed. a-c) immune related pathways enriched for differently expressed genes from IC1, IC3 and IC4, respectively. Significant threshold at $p < 0.05$ are indicated.

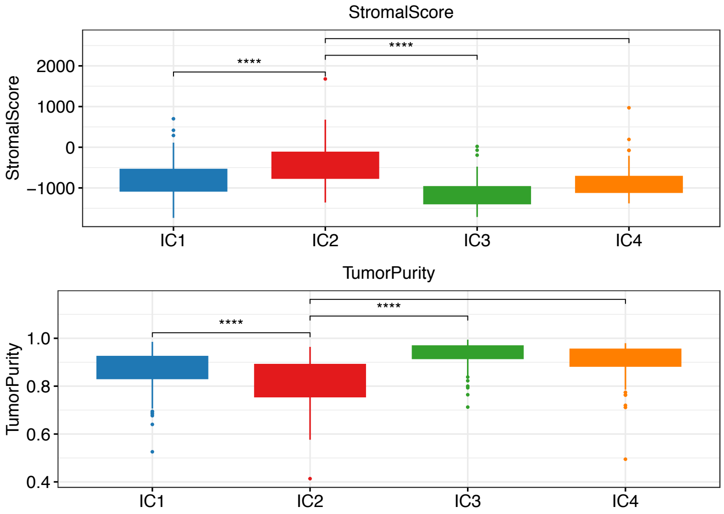

Supplementary Figure 12: Boxplots showing the distribution of ESTIMATE stromal score and tumor purity across different clusters. The IC2 cluster shows height stromal score and lowest tumor purities.

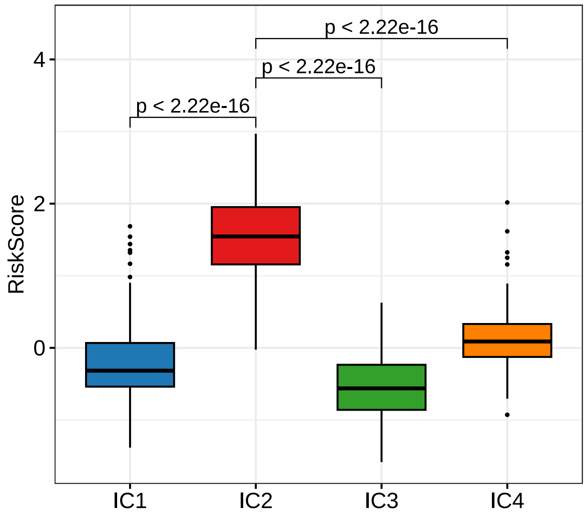

Supplementary Figure 13: Boxplots showing the distribution of Risk score across different clusters. The IC2 cluster shows height risk score.

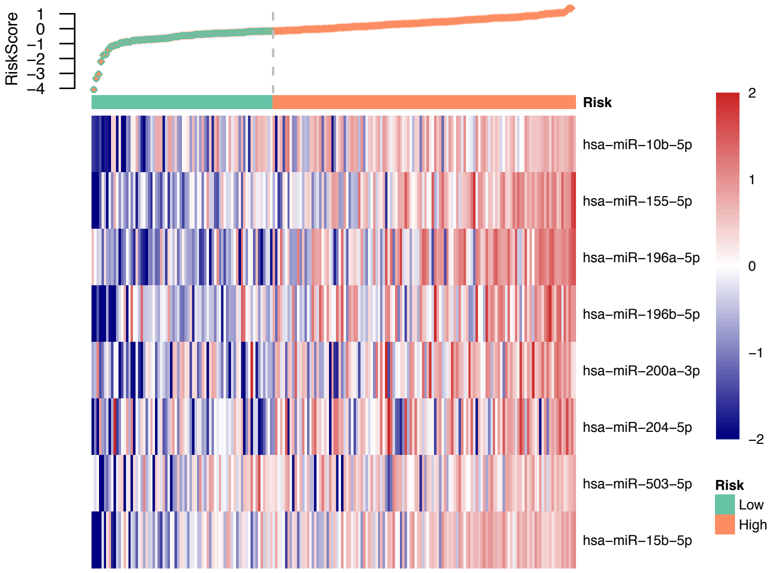

Supplementary Figure 14: Risk score and expression heatmap of the 8 signature miRNAs in the validation set from CGGA.

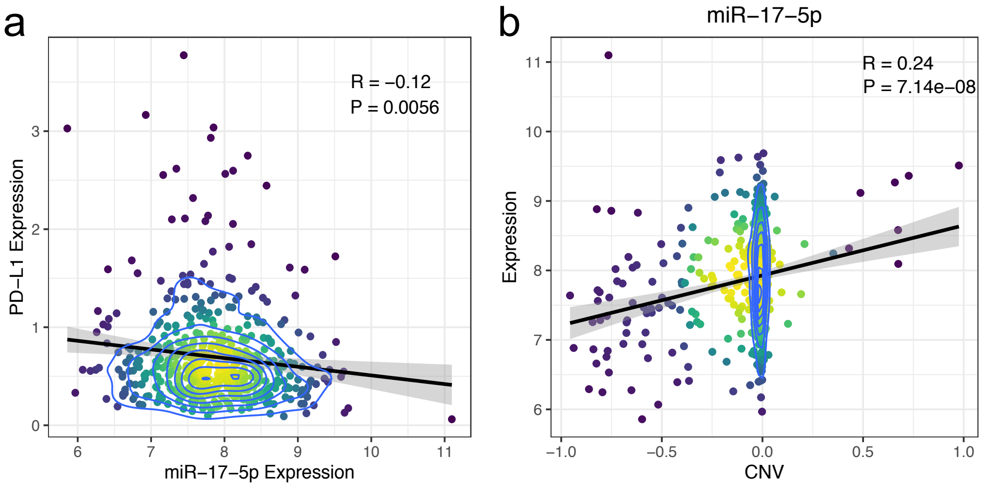

Supplementary Figure 15: Scatter plot showing the significant correlation between expression of PD-L1 and miR-17-5p (a), and correlation of expression and CNV of miR-17-5p (b). The Pearson correlation coefficient and p-values are indicated.

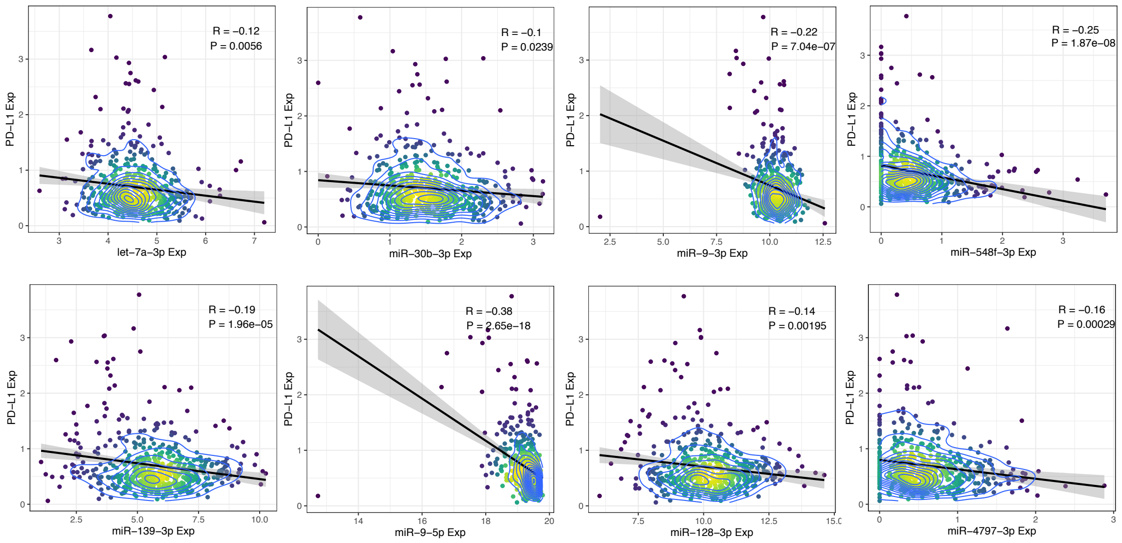

Supplementary Figure 16: Scatter plot showing the significant correlation between expression of PD-L1 and eight miRNAs of let−7a−3p, miR−30b−3p, miR−9−3p, miR−548f−3p, miR−139−3p miR−9−5p, miR−128−3p, miR−4797−3p, respectively. The Pearson correlation coefficient and p-values are indicated.

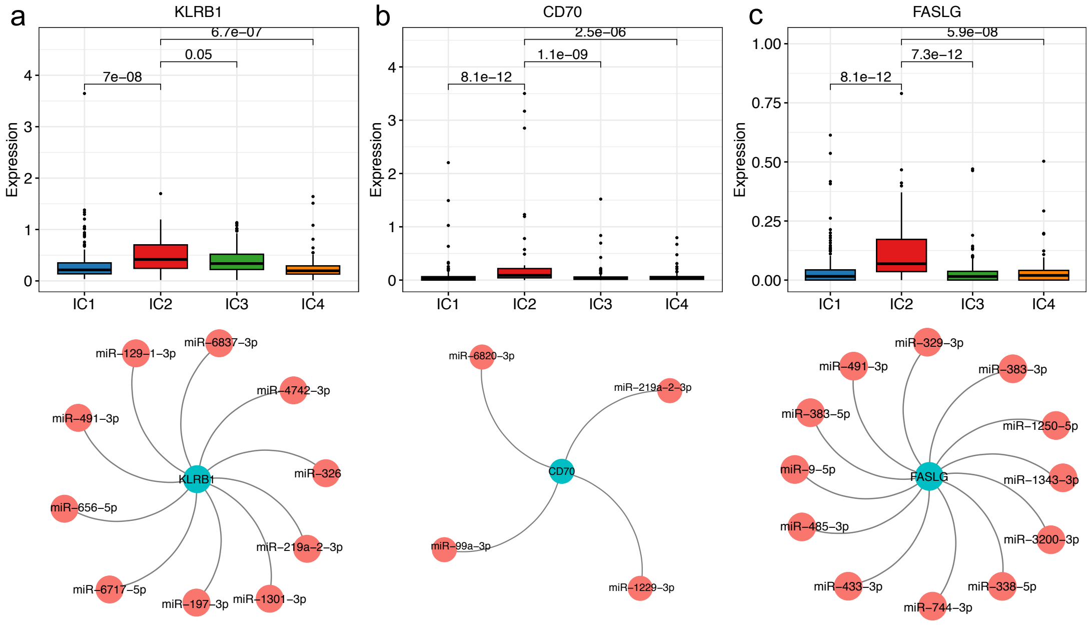

Supplementary Figure 17: Expression distribution of three immune evasion related genes and associated miRNA regulatory modules. a-c). Box plot showing significant elevated expression of KLRB1, CD70 and FASLG in IC2 cluster, the regulatory modules consists of the immune evasion related genes and associated miRNA regulators are also presented.

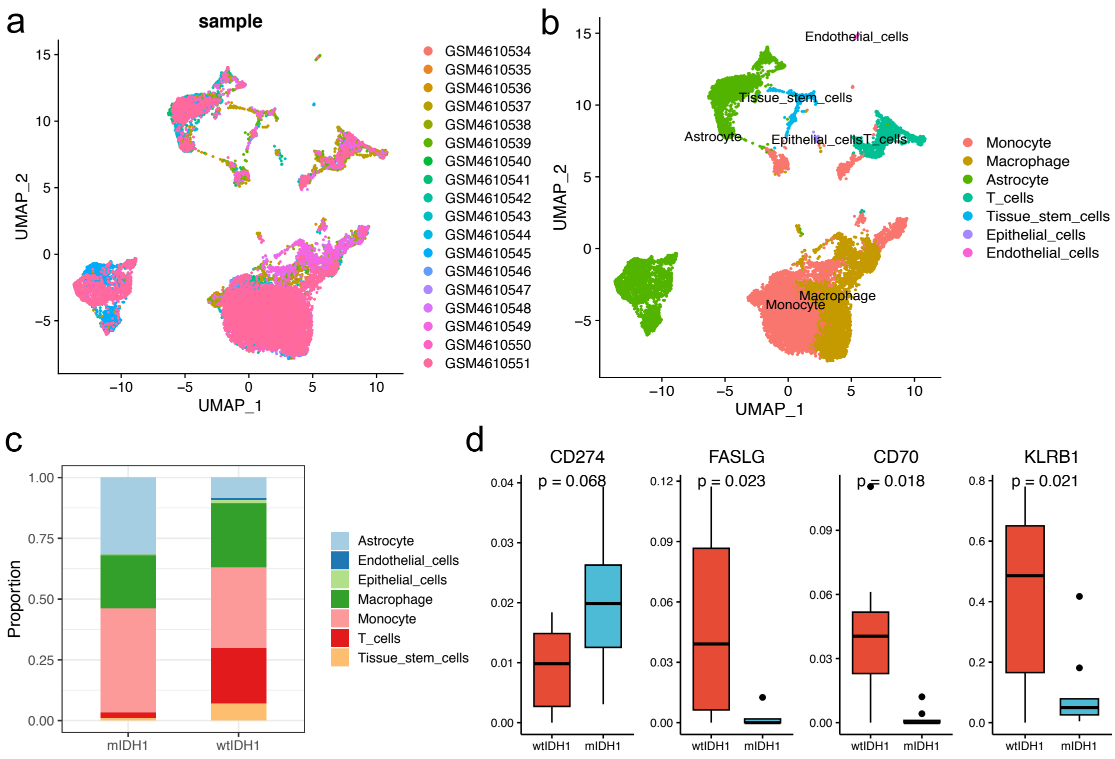

Supplementary Figure 18: Single-cell RNA sequencing (scRNA-seq) analysis of IDH1 wild type (wtIDH1) and mutant (mIDH1) samples in glioma. a). UMAP visualization of 18,260 cells from 18 glioma tumor samples (GSE152273); b). UMAP plot of the seven main cell types identified based on single-cell transcriptomes; c). Relative cell proportion of different cell types in wild type and mutant samples; d). Relative expression of four immune evasion related genes in wild type and mutant samples.

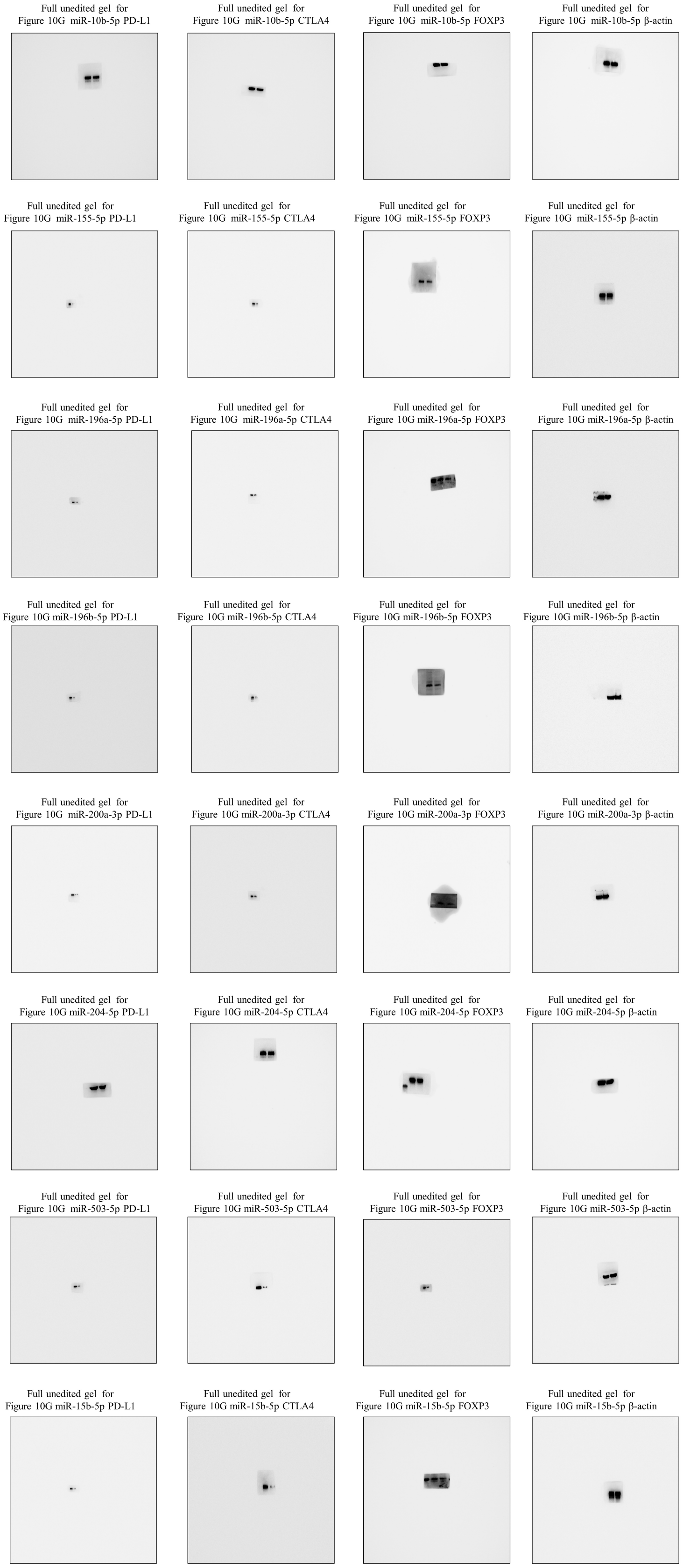

Supplementary Figure 19: Raw images for Western blotting experiments.
