# Supplementary material for: Integrative analysis of genomic and epigenomic regulation reveals miRNA mediated tumor heterogeneity and immune evasion in lower grade glioma
Source: Commun Biol. 2024 Jul 6;7:824. doi: 10.1038/s42003-024-06488-9 (PMC11227553; doi:10.1038/s42003-024-06488-9)
Supplement: Supplementary file 3 — Description of Additional Supplementary Files [file 42003_2024_6488_MOESM3_ESM.pdf]

Description of Additional Supplementary Files

File name: Supplementary Data 1

Description: Genome coordinate of miRNA transcription start site collected from FANTOM5

File name: Supplementary Data 2

Description: Probe information of CpG sites located within miRNA promoters

File name: Supplementary Data 3

Description: Statistics of the 351 miRNAs identified as correlated for their copy number and expression

File name: Supplementary Data 4

Description: Statistics of the 541 miRNAs identified as correlated for their promoter methylation and expression.

File name: Supplementary Data 5

Description: Immune signature gene sets collected from public literatures

File name: Supplementary Data 6

Description: List of the 63 marker genes for 21 immune cell types collected from public resources

File name: Supplementary Data 7

Description: Statistics of the 195 miRNAs identified that correlated with infiltrate level of six immune cell types

File name: Supplementary Data 8

Description: Lists of “Immune Function” and “Cancer Immunology” related miRNAs

File name: Supplementary Data 9

Description: The clinical information of 30 low grade glioma patients of our in-house cohort
