# Supplementary material for: Integrative analysis of genomic and epigenomic regulation reveals miRNA mediated tumor heterogeneity and immune evasion in lower grade glioma
Source: Commun Biol. 2024 Jul 6;7:824. doi: 10.1038/s42003-024-06488-9 (PMC11227553; doi:10.1038/s42003-024-06488-9)
Supplement: Supplementary file 5 — reporting summary [file 42003_2024_6488_MOESM5_ESM.pdf]

nature portfolio

Corresponding author(s): Zhen Yang

Last updated by author(s): Jun 7, 2024

Reporting Summary

Nature Portfolio wishes to improve the reproducibility of the work that we publish. This form provides structure for consistency and transparency in reporting. For further information on Nature Portfolio policies, see our Editorial Policies and the Editorial Policy Checklist.

Statistics

For all statistical analyses, confirm that the following items are present in the figure legend, table legend, main text, or Methods section.

n/a	Confirmed	
☐	☒	The exact sample size (*n*) for each experimental group/condition, given as a discrete number and unit of measurement
☐	☒	A statement on whether measurements were taken from distinct samples or whether the same sample was measured repeatedly
☐	☒	The statistical test(s) used AND whether they are one- or two-sided<br>*Only common tests should be described solely by name; describe more complex techniques in the Methods section.*
☐	☒	A description of all covariates tested
☐	☒	A description of any assumptions or corrections, such as tests of normality and adjustment for multiple comparisons
☐	☒	A full description of the statistical parameters including central tendency (e.g. means) or other basic estimates (e.g. regression coefficient) AND variation (e.g. standard deviation) or associated estimates of uncertainty (e.g. confidence intervals)
☐	☒	For null hypothesis testing, the test statistic (e.g. *F*, *t*, *r*) with confidence intervals, effect sizes, degrees of freedom and *P* value noted<br>*Give P values as exact values whenever suitable.*
☒	☐	For Bayesian analysis, information on the choice of priors and Markov chain Monte Carlo settings
☒	☐	For hierarchical and complex designs, identification of the appropriate level for tests and full reporting of outcomes
☐	☒	Estimates of effect sizes (e.g. Cohen's *d*, Pearson's *r*), indicating how they were calculated

Our web collection on statistics for biologists contains articles on many of the points above.

Software and code

Policy information about availability of computer code

Data collection	No custom code was used to generate data used in this study
Data analysis	R packages and specific functions, as well as softwares used are described in relevant sections in the method section.

For manuscripts utilizing custom algorithms or software that are central to the research but not yet described in published literature, software must be made available to editors and reviewers. We strongly encourage code deposition in a community repository (e.g. GitHub). See the Nature Portfolio guidelines for submitting code & software for further information.

Data

Policy information about availability of data

All manuscripts must include a data availability statement. This statement should provide the following information, where applicable:

- Accession codes, unique identifiers, or web links for publicly available datasets
- A description of any restrictions on data availability
- For clinical datasets or third party data, please ensure that the statement adheres to our policy

Data analyzed in this manuscript is already publicly available from The Cancer Genome Atlas (TCGA) data portal: https://portal.gdc.cancer.gov/ and the Chinese Glioma Genome Atlas (CGGA) data portal: http://www.cgga.org.cn/. The Count matrix of single cell RNA-seq used was obtained from the GEO database with the accession number GSE152273. The source data behind the graphs in the paper is provided in Figshare at: https://doi.org/10.6084/m9.figshare.25989109.v1.

Research involving human participants, their data, or biological material

Policy information about studies with human participants or human data. See also policy information about sex, gender (identity/presentation), and sexual orientation and race, ethnicity and racism.

Reporting on sex and gender	The sex and gender issue was not considered in the design of this study, and the result obtained is not sex and gender specific.
Reporting on race, ethnicity, or other socially relevant groupings	The race and ethnicity issue was not considered in the design of this study, and the result obtained is not race and ethnicity specific.
Population characteristics	The population related issue was not considered in the design of this study, and the result obtained is not population specific.
Recruitment	Human tissue samples of 30 pairs of adjacent non-tumor and glioma samples were collected from the Department of Neuro-surgery of the First Affiliated Hospital, Wannan Medical College (Wuhu, Anhui, P. R. China) and Department of Neuro-surgery of Huashan Hospital, Fudan University (Shanghai, P. R. China) from February 2023 to September 2023. Patients received no systemic treatment before the surgery.
Ethics oversight	All surveys and experiments were approved by the Ethic Committee for Clinical Research of the First Affiliated Hospital of Wannan Medical College and Huashan Hospital, Fudan University, respectively.

Note that full information on the approval of the study protocol must also be provided in the manuscript.

Field-specific reporting

Please select the one below that is the best fit for your research. If you are not sure, read the appropriate sections before making your selection.

☒ Life sciences ☐ Behavioural & social sciences ☐ Ecological, evolutionary & environmental sciences

For a reference copy of the document with all sections, see nature.com/documents/nr-reporting-summary-flat.pdf

Life sciences study design

All studies must disclose on these points even when the disclosure is negative.

Sample size	A total of 500 samples with three types of high-throughput profiling data, which incldue the copy number variation, DNA methylation and miRNA expression were collected from The Cancer Genome Atlas (TCGA), An external dataset of 198 glioma patients with miRNA expression profiling data collected from the Chinese Glioma Genome Atlas (CGGA). 10 IDH1 mutant and 8 IDH1 wild type glioma samples with scRNA-seq data were downloaded from the GEO database (GSE152273)
Data exclusions	No data excluded from the ananlysis
Replication	Human tissue samples of 30 pairs of adjacent non-tumor and glioma were collected for qRT-PCR validation
Randomization	We used the TCGA LGG cohort data as training set and CGGA cohort data as validation set, so no internal randomization was used.
Blinding	not relevant to the study, reasons see above

Reporting for specific materials, systems and methods

We require information from authors about some types of materials, experimental systems and methods used in many studies. Here, indicate whether each material, system or method listed is relevant to your study. If you are not sure if a list item applies to your research, read the appropriate section before selecting a response.

Materials & experimental systems

n/a	Involved in the study	
☐	☒	Antibodies
☐	☒	Eukaryotic cell lines
☒	☐	Palaeontology and archaeology
☒	☐	Animals and other organisms
☒	☐	Clinical data
☒	☐	Dual use research of concern
☒	☐	Plants

Methods

n/a	Involved in the study	
☒	☐	ChIP-seq
☒	☐	Flow cytometry
☒	☐	MRI-based neuroimaging

Antibodies

Antibodies used	anti-PD-L1 (1:1000, Cat#13684; Cell Signaling Technology, Danvers, MA, USA), anti-β-actin (1:1000, Cat#A1978, Sigma-Aldrich, Victoria, BC, Canada), anti-CTLA4 (Cat#53560; Cell Signaling Technology, Danvers, MA, USA), and anti-FOXP3 (1:1000, Cat#12632; Cell Signaling Technology, Danvers, MA, USA)
Validation	All the antibodies were all obtained from Cell Signaling Technology (Danvers, MA, USA).

Eukaryotic cell lines

Policy information about cell lines and Sex and Gender in Research

Cell line source(s)	The human glioma cell line U251 used in this study was purchased from the American Type Culture Collection (ATCC, Manassas, VA, USA).
Authentication	The U251 cell line was characterized by DNA fingerprinting, cell vitality detection, isozyme detection, and mycoplasma detection. The last cell characterization was performed in June 2023
Mycoplasma contamination	All cell lines tested negative for mycoplasma contamination
Commonly misidentified lines (See ICLAC register)	None

Plants

Seed stocks	Not applicable
Novel plant genotypes	Not applicable
Authentication	Not applicable
